# Association Between Hepatobiliary Scintigraphy and Delays in Operative Management in Acute Cholecystitis

**DOI:** 10.7759/cureus.112515

**Published:** 2026-07-12

**Authors:** Shelley Warner, Andy Aleman Espino, Stacey L Tannenbaum, Jahnavi Patel, Rachel Kessler, Gary Lehr

**Affiliations:** 1 General Surgery, Broward Health Medical Center, Fort Lauderdale, USA; 2 Medicine, Dr. Kiran C. Patel College of Osteopathic Medicine, Nova Southeastern University, Fort Lauderdale, USA

**Keywords:** community hospital, hepatobiliary scintigraphy, length of stay, surgical timing, ultrasonography

## Abstract

Background: Ultrasonography (US) is the first-line imaging modality for acute cholecystitis, while hepatobiliary scintigraphy (hepatobiliary iminodiacetic acid, or HIDA, scan) is often reserved for equivocal cases. Despite its diagnostic accuracy, HIDA requires more time and resources and is often an unnecessary and overutilized diagnostic test. Despite significant advancements in medical technology and efficiency, overutilization of these tests continues. This study aimed to determine if HIDA scans continue to cause delays in patient care, with the primary objective being to evaluate the effect of HIDA scans on time to surgery (TTS) and length of stay (LOS).

Methods: We conducted a retrospective cohort study of adult patients admitted through the emergency department to a community hospital, between January 2019 and December 2020, who underwent cholecystectomy. Patients were stratified by preoperative imaging pathway: US alone versus US and HIDA. The primary outcomes were TTS and LOS. Secondary outcomes included complicated gallbladder disease, which was defined by a dilated common bile duct, elevated bilirubin, or elevated lipase indicating choledocholithiasis or gallstone pancreatitis. Independent-samples t-tests were used for group comparisons, and multivariable linear regression was performed to identify predictors of TTS.

Results: Of 159 patients, 84 (52.9%) received US only, and 75 (47.2%) underwent both US and HIDA. Mean TTS was significantly longer in the HIDA group (68.4 vs. 54.1 hours, p=0.039). Excluding patients with complicated gallbladder disease, the difference was more pronounced (59.5 vs. 31.2 hours, p=0.001). LOS did not differ between groups overall (135.4 vs. 133.7 hours, p=0.932) or in uncomplicated cases (126.8 vs. 118.7 hours, p=0.770). In regression analysis, HIDA scan (B=0.885, p=0.001) and elevated bilirubin (B=1.011, p=0.003) were independently associated with increased TTS, while ethnicity (Hispanic, B=-0.804, p=0.021) independently predicted shorter TTS.

Conclusion: In this community hospital cohort, HIDA scans were associated with a significant delay in the operative management of acute cholecystitis by more than 24 hours. Our findings suggest that HIDA scans continue to prolong surgical timing despite evolving hospital workflows and thus, ordering this diagnostic test should be reserved only for equivocal cases.

## Introduction

Gallstone disease is highly prevalent in the United States, with approximately 500,000 cholecystectomies performed annually [1,2]. Optimal diagnosis and management are critical for improving patient outcomes and reducing healthcare costs. Imaging is central to diagnosis, with ultrasonography (US) and hepatobiliary scintigraphy (hepatobiliary iminodiacetic acid (HIDA) scan) being the most utilized modalities. The choice of imaging can directly influence both diagnostic certainty and the timeliness of surgical intervention.

The Tokyo Guidelines recommend US as the first-line imaging modality for acute cholecystitis due to its accessibility, rapid acquisition, portability, and cost-effectiveness [3,4]. Although often the only imaging required for diagnosis with high sensitivity, US does have limitations in accuracy due to user dependence [5]. HIDA scans, on the other hand, demonstrate higher diagnostic accuracy with sensitivities and specificities often exceeding 90% [6-8]. For this reason, HIDA is generally reserved for patients with equivocal clinical or sonographic findings [9-11]. However, HIDA scans are frequently overutilized, even in patients who already meet diagnostic criteria based on clinical presentation, laboratory results, and US [10,12,13].

One key concern with HIDA scans is the substantial additional time required for their completion. While a US can typically be obtained within 30 minutes at any time of day, HIDA scans require a special team that is only available during daytime hours and require a several-hour fasting window in addition to an acquisition time of up to four hours [6,7]. In practice, this delay may defer operative intervention or time to surgery (TTS) until the following day, which may in turn increase length of stay (LOS), particularly in community hospitals where nonemergent procedures are rarely performed at night.

Early operative management of acute cholecystitis decreases morbidity and mortality, conversion from laparoscopic to open surgery, and overall hospital costs [14-16]. While the specific definition of “early” varies across studies, with some supporting surgery within 24 hours of admission [16], others within 48 hours [14], and still others within 72 hours [15], the overall consensus favors operative intervention as soon as feasible once diagnosis is confirmed to optimize outcomes. The last major studies investigating the effect of diagnostic imaging with HIDA scans on TTS and LOS were conducted nearly a decade ago [17]. Given the rapid pace of change in medical practice, it is possible that workflows and hospital resource utilization have evolved, and it is unclear whether HIDA scans continue to cause delays in contemporary settings. With the known overutilization of HIDA scans [12-13], it is important to determine if there are clinical impacts of this overuse.

Few studies have focused specifically on community hospital environments, where workflow and resource allocation differ significantly from academic centers. The primary objective of this study was to determine whether HIDA scan utilization is associated with increased TTS and hospital LOS among patients undergoing cholecystectomy for acute cholecystitis. A secondary objective was to evaluate these associations in a contemporary community hospital setting, where practice patterns may differ from those previously reported in academic institutions. We hypothesized that patients receiving HIDA scans would experience a significant delay in operative intervention compared with those evaluated without HIDA, and that these delays would be associated with increased LOS.

## Materials and methods

Study design 

This was a retrospective cohort study of patients admitted to one community hospital with gallbladder disease. Institutional review board exemption was obtained before study initiation due to the retrospective nature of the study and the low risk of loss of confidentiality to the patients. Electronic medical records were reviewed manually by the study investigators, and demographic, laboratory, imaging, operative, and hospitalization variables were abstracted into a standardized database for analysis. Patients admitted through the emergency department between January 1, 2019, and December 31, 2020, who were 18 years or older and received a gallbladder US and/or HIDA scan were included. Patients were excluded if they did not undergo a cholecystectomy during the same hospital admission as the imaging.

The primary outcome was TTS, defined as hours from hospital admission to initiation of surgery in the operating room. Secondary outcomes included hospital LOS, defined as hours from admission to discharge, and complications of gallbladder disease, defined as diagnosis of choledocholithiasis and gallstone pancreatitis. Imaging findings were obtained from formal radiology reports interpreted according to routine institutional practice. Choledocholithiasis was considered as ductal dilation read by a radiologist on a US or CT scan (>7 mm) or an elevated bilirubin (>1.5 mg/dL). Gallstone pancreatitis was characterized by an elevated lipase (>80 U/L). The two comparison groups consisted of patients who underwent preoperative imaging with a HIDA scan in addition to US versus no HIDA (US ± CT only). Demographic information for each patient was recorded from the electronic medical record (EMR), including age (years), sex (male/female), race (Black or African American, White, other), and ethnicity (Hispanic/non-Hispanic). Demographic and clinical variables collected included body mass index (BMI, kg/m²), diabetes mellitus (yes/no), and elevated liver enzymes (aspartate aminotransferase, alanine transaminase, and/or alkaline phosphatase), the latter serving as a potential marker of gallbladder inflammation severity.

Statistical analysis

Demographic data and other characteristics of patients were presented as means and standard deviations (SDs). Categorical data were expressed as frequencies and percentages. Chi-square or Fisher’s exact test (as applicable) was used to compare associations of categorical variables to one another. Continuous variables were compared between patients with and without HIDA scans using two-sided independent-samples t-tests. A multivariable linear regression model was constructed to evaluate predictors of TTS. Variables entered into the model were selected based on clinical relevance and univariable associations with TTS and included age, sex, race, ethnicity, BMI, ductal dilation, lipase, bilirubin, gallbladder pathology, elevated liver enzymes, HIDA scan utilization, and length of stay. A significance level of 0.05 was used for all tests. Data analysis was performed using IBM SPSS Statistics for Windows, version 30 (released 2023; IBM Corp., Armonk, USA).

## Results

A total of 1,000 patients were admitted to the community hospital during the study period; these patients underwent gallbladder imaging with US or CT with or without HIDA scanning, and subsequently underwent cholecystectomy. Given the retrospective design and labor-intensive chart abstraction, a random computer-generated sample of 260 eligible patients was initially selected for review. After applying exclusion criteria, 159 patients remained. Following completion of chart abstraction for these patients, a post hoc analysis demonstrated statistical significance in the primary outcome of time to surgery; therefore, no additional charts were reviewed, and these 159 patients comprised the final analytic cohort. In the final cohort, all patients underwent laparoscopic or robotically assisted laparoscopic cholecystectomy without conversion to an open procedure.

The mean age of the total sample was 52.9 years (SD 18.4), with a mean BMI of 30.5 kg/m² (SD 7.4) (Table 1). The majority of patients were female (60%), White (70%), and non-Hispanic (77%), and did not have diabetes (84%). No significant demographic differences were observed between patients who underwent HIDA scans and those who did not. No mortality was observed in our sample, nor were there any significant complications recorded in the initial hospitalization. US was used as the initial imaging except for three patients who underwent a CT scan without an ultrasound; these were included in the analysis as the no HIDA group. This small subgroup (1.9% of the sample) was not large enough to bias the US alone versus HIDA comparison.

**Table 1:** Demographic data for all patients and between cohorts based on imaging modality Data are presented as means ± standard deviations (SDs) for continuous variables and n (%) for categorical variables. Continuous variables were compared using two-sided independent-samples t-tests with unequal variances. Categorical variables were compared using Pearson chi-square tests. Test statistics and corresponding p-values are reported. Statistical significance was defined as p<0.05. *t-statistic from the two-sided independent-samples t-test with unequal variances. ^Pearson chi-square statistic. n/a: not applicable; HIDA scan: hepatobiliary iminodiacetic acid scan

Characteristics	All patients (n=159), n (%)	HIDA (n=75), n (%)	No HIDA (n=84), n (%)	Test statistic	p-value
Age, mean (SD)	52.9 (18.4)	52.2 (17)	53.9 (19)	1.975^*^	0.68
BMI, mean (SD)	30.5 (7.4)	30.6 (8.2)	27.8 (6.9)	1.977^*^	0.738
Sex, n (%)				1.841^^^	0.175
Male	64 (40)	26 (35)	38 (45)	n/a	n/a
Female	95 (60)	49 (65)	46 (55)	n/a	n/a
Race, n (%)				0.719^^^	0.702
Black	44 (28)	22 (29)	22 (26)	n/a	n/a
White	112 (70)	51 (68)	61 (73)	n/a	n/a
Other	3 (2)	2 (3)	1 (1)	n/a	n/a
Ethnicity, n (%)				0.029^^^	0.865
Hispanic	37 (23)	17 (23)	20 (24)	n/a	n/a
Non-Hispanic	122 (77)	58 (77)	64 (76)	n/a	n/a
Diabetes, n (%)				0.020^^^	0.887
Yes	24 (15)	11 (15)	13 (15)	n/a	n/a
No	135 (84)	64 (85)	71 (85)	n/a	n/a

**Figure 1:**
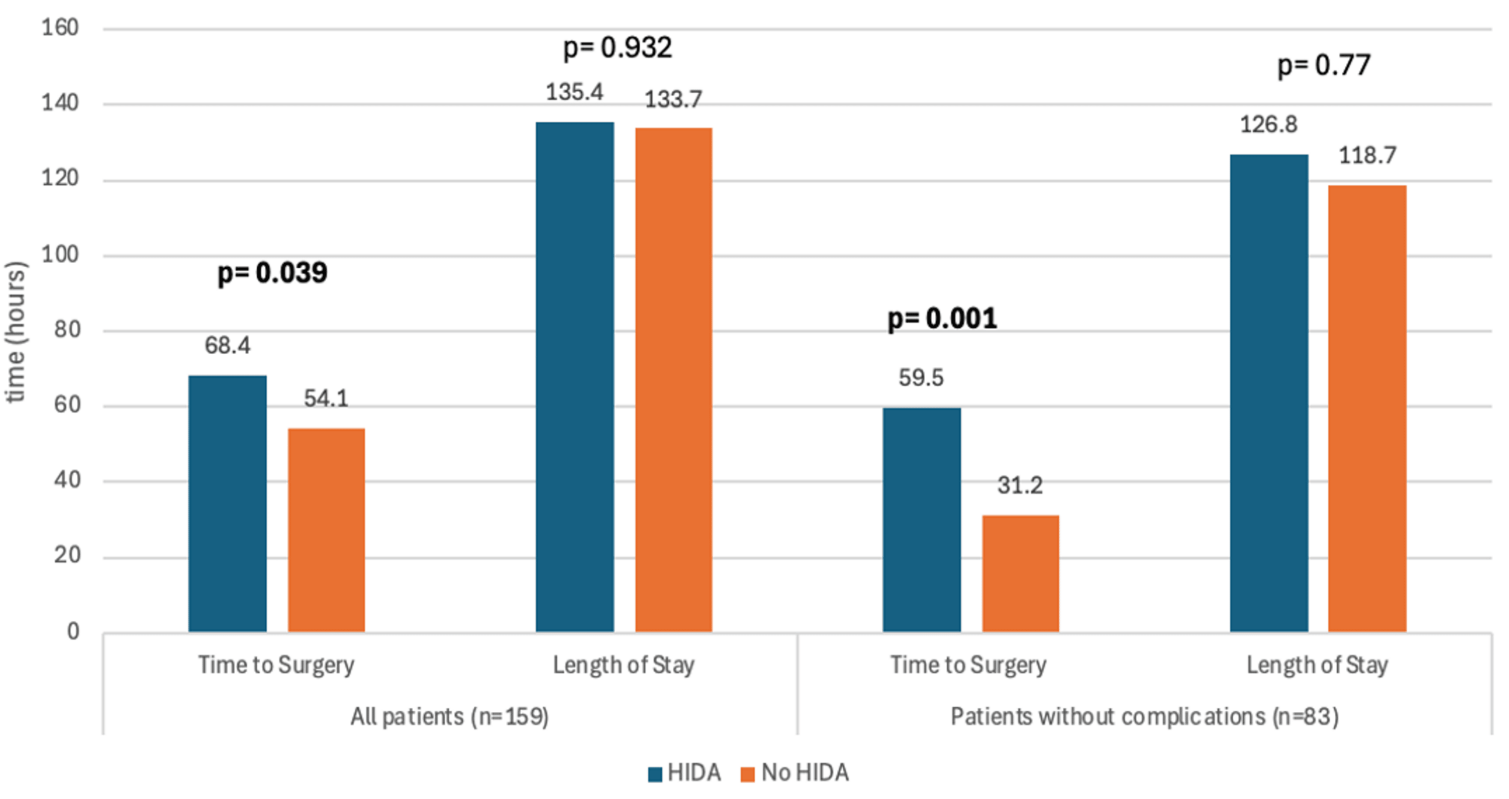
Length of stay and time to surgery based on performing HIDA scans Data are presented as mean hours. Comparisons between groups were performed using two-sided independent-samples t-tests with unequal variances. Corresponding p-values are displayed above each comparison. Statistical significance was defined as p<0.05. Boldfaced p-values indicate statistically significant differences between groups. HIDA scan: hepatobiliary iminodiacetic acid scan

The differences in TTS and LOS with and without HIDA scans are displayed in Table 2 and Figure 1. For all patients (n=159), the mean TTS was 68.4 hours in the HIDA group and 54.1 hours in the no-HIDA group, a statistically significant difference (p=0.039). Among patients without complications of choledocholithiasis and/or gallstone pancreatitis (n=83), the mean TTS was 59.5 hours in the HIDA group versus 31.2 hours in the no-HIDA group (p=0.001), representing a difference of more than 24 hours. Mean values for LOS were similar between groups: 135.4 hours with HIDA versus 133.7 hours without HIDA (p=0.932). In the subgroup without complications, LOS was 126.8 hours for the HIDA group and 118.7 hours for the no-HIDA group, which was also not statistically significant (p=0.770).

**Table 2:** Length of stay and time to surgery based on whether a HIDA scan was done or not Data are presented as mean hours. Comparisons between groups were performed using two-sided independent-samples t-tests with unequal variances assumed. t-statistics and corresponding p-values are reported. Statistical significance was defined as *p<0.05 and ** p<0.01. HIDA scan: hepatobiliary iminodiacetic acid scan; TTS: time to surgery; LOS: length of stay

	TTS (hours)		t-statistic	p-value	LOS (hours)		t-statistic	p-value
	HIDA	No HIDA			HIDA	No HIDA		
All patients (n=159)	68.4	54.1	2.10	0.039*	135.4	133.7	0.14	0.932*
Patients without complications (n=83)	59.5	31.2	3.75	0.001**	126.8	118.7	0.20	0.770**

In univariate linear regression analyses (Table 3), several variables were significantly associated with time to surgery. Patients who underwent HIDA scans experienced longer TTS compared with those who did not (B=0.597, 95% CI 0.032-1.162, p=0.039). Elevated bilirubin (B=1.249, 95% CI 0.689-1.809, p<0.001), elevated liver enzymes (B=0.867, 95% CI 0.307-1.426, p=0.003), and gallbladder pathology (B=0.343, 95% CI 0.032-0.654, p=0.031) were also associated with increased time from admission to operation. Longer length of stay correlated strongly with longer TTS (B=0.005, 95% CI 0.003-0.007, p<0.001). Conversely, higher BMI was associated with shorter TTS (B=−0.041, 95% CI −0.079 to −0.002, p=0.037), and Hispanic ethnicity predicted faster progression to surgery (B=−0.734, 95% CI −1.401 to −0.068, p=0.031). Ductal dilation demonstrated a trend toward significance (p=0.059), while age, sex, race, and lipase were not significant predictors.

**Table 3:** Univariable and multivariable linear regression for predictors of TTS Results are presented as unstandardized regression coefficients (B), 95% confidence intervals (CIs), and p-values. Univariable and multivariable linear regression models were used to evaluate associations between clinical variables and TTS. Statistical significance was defined as p<0.05. Overall model fit: F(12,128) = 6.54, p<0.001; R²=0.380; adjusted R²=0.322. *Significant at <0.05; **significant at <0.01; ***significant at <0.001. ^a^Aspartate aminotransferase (AST), alanine transaminase (ALT), alkaline phosphatase (ALP) HIDA scan: hepatobiliary iminodiacetic acid scan; TTS: time to surgery; LOS: length of stay; GB: gallbladder

	Univariable			Multivariable		
Predictor	B	95% CI	p-value	B	95% CI	p-value
HIDA	0.597	(0.032, 1.162)	0.039*	0.885	(0.350, 1.421)	0.001**
Bilirubin	1.249	(0.689, 1.809)	<0.001***	1.011	(0.345, 1.676)	0.003**
Ethnicity (Hispanic)	−0.734	(−1.401, −0.068)	0.031*	−0.804	(−1.484, −0.124)	0.021*
LOS (hours)	0.005	(0.003, 0.007)	<0.001***	0.005	(0.003, 0.008)	<0.001***
BMI	−0.041	(−0.079, −0.002)	0.037*	−0.030	(−0.066, 0.006)	0.103
Ductal dilation	0.714	(−0.028, 1.456)	0.059	0.216	(−0.475, 0.907)	0.537
Elevated enzymes (any)^a^	0.867	(0.307, 1.426)	0.003**	0.363	(−0.300, 1.025)	0.281
GB pathology	0.343	(0.032, 0.654)	0.031*	0.126	(−0.170, 0.423)	0.401
Sex	0.156	(−0.426, 0.739)	0.597	0.450	(−0.113, 1.012)	0.116
Race	0.216	(−0.351, 0.783)	0.454	−0.097	(−0.665, 0.472)	0.737
Lipase	0.598	(−0.184, 1.381)	0.133	0.083	(−0.605, 0.771)	0.812
Age	0.011	(−0.005, 0.028)	0.175	0.011	(−0.005, 0.028)	0.175

When all significant and clinically relevant variables were entered into a multivariate linear regression model, the overall model was statistically significant (F(12,128) = 6.54, p<0.001), explaining 38% of the variance in TTS (R²=0.380, adjusted R²=0.322). After adjusting for demographic and clinical covariates, HIDA scan remained an independent predictor of delayed surgery (B=0.885, 95% CI 0.350-1.421, p=0.001). Elevated bilirubin also independently predicted longer TTS (B=1.011, 95% CI 0.345-1.676, p=0.003), whereas Hispanic ethnicity remained associated with earlier surgery (B=−0.804, 95% CI −1.484 to −0.124, p=0.021). Additionally, LOS remained positively correlated with TTS (B=0.005, 95% CI 0.003-0.008, p<0.001). Other variables, including age, sex, BMI, race, ductal dilation, lipase, gallbladder pathology, and elevated liver enzymes, were not statistically significant predictors after adjustment.

## Discussion

The purpose of this study was to evaluate whether the use of HIDA scans continues to delay time to surgery and increase hospital length of stay for patients undergoing cholecystectomy for acute cholecystitis, with a focus on a community hospital setting. Our results demonstrate that HIDA scans were independently associated with a significant delay in TTS of more than 24 hours, even after adjusting for demographic and clinical covariates. Importantly, elevated bilirubin was also an independent predictor of delayed surgery due to the potential need for additional imaging or procedure before an operation. Hispanic ethnicity was associated with earlier operative intervention. Despite these differences in TTS, the overall LOS did not differ significantly between groups.

These findings reinforce the Tokyo Guidelines, which recommend US as the first-line imaging modality for suspected acute cholecystitis due to its accessibility, rapid acquisition, and cost-effectiveness [3,4]. While HIDA scans have superior diagnostic accuracy compared to US [6-9], they introduce significant workflow challenges due to the length of the study itself and the inability to perform it overnight. In our study, HIDA use was associated with nearly one additional day of delay in proceeding to surgery, consistent with prior reports [17]. Importantly, our regression analysis confirmed that this association persisted after adjusting for other risk factors, underscoring HIDA as an independent driver of surgical delay.

Our findings update a literature base that has remained largely unchanged for nearly a decade. Previous studies reported that HIDA scans prolonged TTS and sometimes LOS [11,17], but most were conducted in academic or for-profit hospitals. Given the rapidly evolving nature of medical practice, it was unclear whether these delays persisted in modern workflows. Our results suggest that despite advances in diagnostics and hospital processes, the impact of HIDA scans on operative timing remains clinically meaningful in community hospital environments.

While LOS was not significantly different between groups, this finding may reflect hospital workflow and patient factors rather than the absence of clinical impact. Patients who experienced preoperative delays may have been more eager for expedited discharge after surgery, mitigating the effect on total LOS. Alternatively, it is possible that our sample size limited our ability to detect a modest LOS difference. Other institutions, particularly those with “delayed” HIDA protocols, have reported longer hospitalizations [11,17].

As expected, complicated gallbladder disease increased the time to surgery, and when excluded, demonstrated an even bigger difference between the HIDA group and the no HIDA group. Elevated bilirubin independently predicted longer TTS, consistent with suspected choledocholithiasis and the possibility of undergoing additional imaging or procedure, endoscopic retrograde cholangiopancreatography, before operative intervention for the gallbladder. However, downstream procedures such as magnetic resonance cholangiopancreatography (MRCP), endoscopic retrograde cholangiopancreatography (ERCP), or intraoperative cholangiography were not systematically recorded and therefore could not be analyzed. Interestingly, the elevated lipase as a marker of gallstone pancreatitis did not demonstrate a significant effect of TTS, as patients can often recover from the pancreatitis quickly without further intervention before their cholecystectomy. Additionally, patients with severe pancreatitis are often given a longer time to recover from the pancreatitis, and their cholecystectomy is done on a different admission and would not have been included in this cohort.

Hispanic ethnicity independently predicted shorter time to surgery in our multivariable analysis. This finding was unexpected, as the existing literature on healthcare disparities often demonstrates delayed access to care among minority populations [18,19]. Because ethnicity was not a primary variable of interest in this study, our analysis was not designed to identify the underlying cause of this association, and any explanation remains speculative. One possible explanation is that the demographic composition of the community served by our institution may influence baseline presentation patterns, provider familiarity with the patient population, or local care pathways. Another possibility is that Hispanic patients in our cohort may have presented with more severe or clinically apparent disease, prompting earlier operative intervention, though severity at presentation was not specifically evaluated in this analysis. Additional unmeasured factors, such as language concordance, family support systems, insurance status, or other social determinants of health, may have also influenced surgical timing. Given the unexpected nature of this finding, further prospective studies are warranted to better understand the relationship between ethnicity and access to timely operative care in community hospital settings.

From a clinical perspective, the evidence remains strong that early cholecystectomy improves outcomes. Large registry data [14], prospective studies [15], and randomized controlled trials [16] have all shown that earlier intervention reduces conversion to open procedures, bile duct injuries, complications, and cost. Definitions of “early” vary - within 24 hours [16], 48 hours [14], or 72 hours [15] - but consensus supports surgery as soon as feasible. Our findings suggest that HIDA scans continue to be a barrier to achieving this goal in community hospital practice.

This study is not without limitations. First, we were unable to directly assess postoperative complications, an important clinical consequence of delayed intervention, due to the relatively small sample size and the absence of major perioperative complications in our cohort. Second, because chart abstraction was discontinued after a post hoc analysis demonstrated statistical significance in the primary outcome, the final sample size was not determined by an a priori power calculation. As a result, the estimated magnitude of effect may be less precise than if the entire eligible cohort had been analyzed. Third, this was a single-institution study, and practice patterns and resource availability in a community hospital may differ from those in other community settings or academic centers, which may limit broader applicability. Additionally, provider-level variables, including surgeon-specific practice patterns and the identity of the ordering clinician, were not collected, and therefore their contribution to delays in operative intervention could not be assessed. Furthermore, the specific clinical rationale for obtaining a HIDA scan was not systematically recorded. Consequently, this study was not designed to determine the appropriateness of HIDA utilization or to distinguish whether HIDA scans were obtained because of equivocal findings, atypical presentations, or provider preference. However, a key strength of this study is its focus on a community hospital environment. Because community hospitals represent the majority of hospitals in the United States, our findings likely reflect common practice and may be more generalizable to similar institutions than prior studies conducted primarily in academic or tertiary care centers.

## Conclusions

In this community hospital cohort study, HIDA scans were independently associated with significant delays in operative management of acute cholecystitis by over 24 hours without effect on LOS. Nearly a decade after prior reports of HIDA scans affecting length of stay, these findings suggest that HIDA scans continue to prolong surgical interventions despite advances in hospital workflows. Given the established benefits of early surgery and overutilization of HIDA scans, it is important to reiterate that HIDA should be reserved for patients with equivocal findings on their workup. US, combined with clinical and laboratory data, should remain the cornerstone of diagnosis for acute cholecystitis.
